# The Distal Free Achilles Tendon Is Longer in People with Tendinopathy than in Controls: A Retrospective Case-Control Study

**DOI:** 10.1155/2022/6585980

**Published:** 2022-08-28

**Authors:** Joanne H. Callow, Mark Cresswell, Faraz Damji, Joshua Seto, Antony J. Hodgson, Alex Scott

**Affiliations:** ^1^Department of Rehabilitation Sciences, University of British Columbia, Vancouver, Canada; ^2^Department of Radiology, University of British Columbia, Vancouver, Canada; ^3^Department of Medicine, University of British Columbia, Vancouver, Canada; ^4^Department of Cellular and Physiological Sciences, University of British Columbia, Vancouver, Canada; ^5^Department of Mechanical Engineering, University of British Columbia, Vancouver, Canada; ^6^Department of Physical Therapy, University of British Columbia, Vancouver, Canada

## Abstract

**Objectives:**

The free Achilles tendon is defined as the region of tendon distal to the soleus which is “unbuttressed,” i.e., unsupported by muscular tissue. We reasoned that a relative lack of distal buttressing could place the tendon at a greater risk for developing Achilles tendinopathy. Therefore, our primary goal was to compare the free Achilles tendon length between those with midportion or insertional Achilles tendinopathy and healthy controls.

**Design:**

This is a retrospective case-control study. *Setting*. Hospital in Vancouver, Canada. *Participants*. 66 cases with Achilles tendinopathy (25 insertional, 41 midportion) consecutively drawn from a hospital database within a 5-year period and matched to 66 controls (without tendinopathy) based on sex, age, and weight. *Main outcome measures*. Odds ratio of the risk of developing Achilles tendinopathy given the length of free tendon, defined anatomically on MRI, after adjustment for confounders.

**Results:**

MRI-defined free Achilles tendon length is a statistically significant predictor of having midportion Achilles tendinopathy (odds ratio = 0.53, 95% confidence interval 1.13 to 2.07). Midportion Achilles tendinopathy cases had significantly longer free tendons (*Mdn* = 51.2 mm, *IQR* = 26.9 mm) compared to controls (*Mdn* = 40.8 mm, *IQR* = 20.0 mm), *p* = 0.007. However, there was no significant difference between the free Achilles tendon lengths in insertional AT cases (*Mdn* = 47.9 mm, *IQR* = 15.1 mm) and controls (*Mdn* = 39.2 mm, *IQR* = 17.9 mm), *p* = 0.158. Free Achilles tendon length was also correlated with the tendon thickness among those with Achilles tendinopathy, *r*_*τ*_ = 0.25, and *p* = 0.003.

**Conclusions:**

The MRI-defined length of the free Achilles tendon is positively associated with the risk of midportion Achilles tendinopathy. A relative lack of distal muscular buttressing of the Achilles tendon may therefore influence the development of tendinopathy.

## 1. Introduction

Achilles tendinopathy is an injury that can cause pain and structural deficits in the tendon and impair the function of the kinetic chain during walking, running, or jumping [1]. It can occur around the enthesis (insertional Achilles tendinopathy) or along the body of the tendon (midportion Achilles tendinopathy). Tendinopathy is thought to occur when the load applied to the tendon exceeds its ability to adapt or repair [2]. This imbalance between load and adaptation or repair can result in cumulative pathological changes within the tendon and its supporting connective tissues. This process is theorized to be a reversible continuum in the early stages, but causing irreversible tendon degeneration in the later stages [3, 4]. For this reason, prevention is a key to prevent long-term suffering from this injury.

In order to prevent a disorder, it is essential to understand who is at risk and what puts them at risk. Some established risk factors for Achilles tendinopathy include age, changes in loading, gender, excessive weight, diabetes, steroid exposure, cholesterol level, recent injury, footwear, and activity level [5]. However, limited research has been found focusing on the potential anatomical risk factors for Achilles tendinopathy. Understanding the anatomy of the muscle-tendon unit is important in understanding the aetiology of injury. Previously, a case-control study has identified, using ultrasound imaging, that the abductor hallucis and flexor digitorum brevis are on average thicker in people with Achilles tendinopathy than in controls, but that the flexor hallucis brevis is thinner [6]. Another case-control study using ultrasound identified a smaller pennation angle in the gastrocnemius muscle of patients with Achilles tendinopathy compared to controls [7]. These observations may be useful when designing rehabilitation programs after the condition has developed, but because muscle thickness and pennation angle are plastic features, it cannot be concluded from a case-control study whether such differences represent risk factors for the development of pathology, or post-injury adaptations.

Conversely, the length of the Achilles tendon is not expected to change significantly during adulthood [8] and may therefore represent a relatively non-plastic feature which, if differing between cases and controls, could be interpreted as a potential risk factor for the development of pathology. The Achilles tendon is divided into three sub-tendons that intertwine as they descend toward the calcaneus [9] Two of the sub-tendons originate from the lateral and the medial heads of the gastrocnemius muscle and are buttressed by the soleus muscle which lies anterior to them. The third sub-tendon, originating from the soleus muscle, joins the other two sub-tendons in the distal portion of the tendon. This section from the distal tip of the soleus to the calcaneus insertion is known as the free Achilles tendon because it has no inserting muscle fascicles and is made up purely of connective tissue [10]. Using magnetic resonance imaging (MRI), the structural differences can be investigated between those with and without tendinopathy [11, 12]. It has been well established that those with tendinopathy have tendons with significantly larger cross-sectional area and anterior-posterior diameter compared to healthy controls [7, 13–26]. This is thought to be mainly due to the increased fluid content and disordered collagen structure as an adaptive and/or reparative response [27–35].

**Figure 1:**
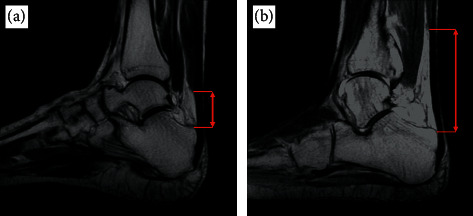
Free Achilles tendon length differences on sagittal T1-weighted MR images. (a) A patient with a short free Achilles tendon (short red arrow). (b) A patient with a long free Achilles tendon (long red arrow).

The length of the free Achilles tendon varies greatly between individuals (Figure 1). Devaprakash et al. reported the free Achilles tendon lengths of 10 different studies showing an average length ranging from 42.8 to 73.7 mm and standard deviation ranging from 7.9 to 19.9 mm [36]. An experienced radiologist with Providence Healthcare has noticed a trend of longer free Achilles tendons in his tendinopathy patients (M. Cresswell, personal communication, September 18, 2020). We reasoned that individuals with a longer free Achilles tendon, i.e., a larger section of distal tendon which is not directly buttressed by the soleus muscle, may be more prone to injury. Therefore, the purpose of this study was to test the hypothesis that the free distal Achilles tendon length will differ between patients with and without Achilles tendinopathy.

## 2. Methods

### 2.1. Study Design

This study used a matched case-control method. Ankle MRI scans from a hospital in Vancouver were identified from January 2015 to December 2019. The patients were divided into those with midportion and insertional Achilles tendinopathy and matched to those without tendinopathy. MRI scans were analyzed to compare the differences between the two study groups. This study received institutional approvals from the University of British Columbia Clinical Research Ethics Board and the Providence Health Care Research Institute. Patient's consent was waived for this retrospective study.

### 2.2. Participants

Patients were included if they had an MRI scan of the left or right ankle at a specific hospital between 2015 and 2019 and were above the age of 18. In order to avoid enrolling patients whose tendinopathy was secondary to a pre-existing condition, patients were excluded if they were pregnant, had systemic inflammatory disorders, had dyslipidemia, or had diabetes [5]. Patients were also excluded if they had ankle surgery or a complete or partial Achilles tendon rupture as these factors may affect the length of the tendon. The case group was composed of those with a diagnosis of midportion or insertional Achilles tendinopathy. The control group was composed of patients who had grossly normal Achilles tendons, and who were evaluated for acute injuries such as sprains, bone contusions, and osteochondral fractures. Controls were excluded if they had any chronic ankle injury (e.g., arthritis, plantar fasciitis, bursitis). Controls were matched to cases based on age, sex, and weight, variables suspected to be correlated with Achilles tendinopathy [5]. The “MatchIt” package was used to match cases to controls [37, 38]. Sex was matched by exact matching, while age and height used Mahalanobis distance matching in order to maximize included case numbers while maintaining covariate proximity [39].

### 2.3. Data Sources

Cases were found using a search of the hospital's electronic medical database for the search term “Achilles tendinopathy.” These patients were then screened by reading the patient medical records and consulting with a radiologist to determine if they had Achilles tendinopathy and which type it was (midportion or insertional). Controls were found using a retrospective review of all patients in the electronic medical database with an MRI requisition. Each patient file was reviewed to determine if the patient matched the eligibility requirements. DICOM MR images were downloaded from the hospital using the SapienSecure deidentifying software [38]. All scans were performed on a 1.5 Tesla MRI system. T1 Axial, coronal, and sagittal images were obtained of the foot and ankle using slice thickness of 3–4 mm, an echo time of 22–42 ms, and a repetition time of 385–732 ms.

### 2.4. Measurement

MRI analysis was performed by three university students using 3D Slicer (http://www.slicer.org) [40]. Data collectors were not told which patients were cases and which were controls; however, blinding was not entirely possible because the AT cases usually demonstrate fusiform thickening which was clearly visible. Each variable was recorded twice and the mean of the measurements was reported. Rater 1 completed the measurements for 33 patients for all MRI outcomes. Rater 2 completed the measurements for 31 different patients for all MRI outcomes. Rater 3 completed the measurements for all remaining patients and repeated them for all patients. Raters were blinded to one another's measurements until the end of the study. The time interval between repeated measurements was a minimum of 48 hours [41].

**Figure 2:**
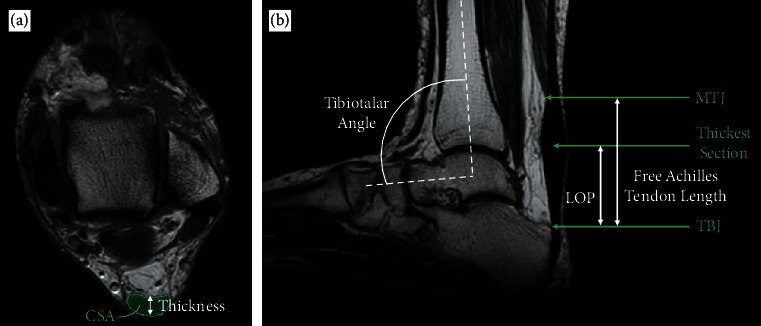
T1-weighted MRI scan demonstrating the measurements made. (a) The axial image with the cross-sectional area (green) and thickness (white arrow) of the tendon. (b) The sagittal image showing the three locations that the cross-sectional area (CSA) and thickness measurements were made (green arrows) as well as the location of pathology (LOP), free Achilles tendon length and tibiotalar angle measurements (white arrows/curve).

The Achilles tendon was segmented at the myotendinous junction, the tendon-bone junction and at the thickest section of the tendon, by manually painting the area of the tendon and paratenon in each axial image slice (Figure 2(a)). The myotendinous junction was identified as the most distal end of the soleus, where the soleus and Achilles tendon meet. The tendon-bone junction was identified as the location the Achilles tendon reaches the superior aspect of the calcaneus. The thickest section of the tendon was defined as the slice with the largest anteroposterior width. 3D Slicer's “Segment cross section area” module calculated the cross-sectional areas of the segmented slices. 3D Slicer's “Segment Statistics” module was used to produce a centroid point for each segmentation. The thickness of the tendon was measured perpendicular to the longest distance across the tendon, through the centroid point from the most posterior point to the most anterior point of the tendon (Figure 2(a)). The distance between the myotendinous junction and tendon-bone junction was measured and defined as the free Achilles tendon length (Figure 2(b)). The location of pathology was defined as location where the Achilles tendon is the thickest and determined by measuring the length between the centroid of the segmented tendon-bone junction slice and the thickest slice (Figure 2(b)). The position of the ankle was assessed by measuring the tibiotalar angle, defined using a technique described by Russel et al. as the angle between the tibial shaft axis and the collum tali axis [42] The tibial shaft axis was found by drawing the centerline of the diaphysis [43]. The collum tali axis was found by drawing the line that bisects the head and neck of the talus [44]. Slicer's “angle markup” tool was used to measure the angle (Figure 2(b)).

### 2.5. Statistical Methods

All statistical analyses were performed using RStudio [45]. Data was checked for missing values, which were dealt with by pairwise deletion and reported in the results section. Descriptive statistics were calculated for the case and control groups and reported as medians with interquartile ranges for continuous variables and number of cases with percentages for categorical variables. Differences between groups were evaluated using a two-stage procedure [46] First, the normality was assessed using the Shapiro-Wilk test. Then, the appropriate statistical test was selected. If the normality test was not significant, a Student's t-test was used. If the normality test was significant, a non-parametric test was used (Wilcoxon rank sum test for continuous variables and Chi-squared test for categorical variables). A *p*-value of less than 0.05 was considered significant. Intra- and interobserver reliability was assessed by calculating the multiple measurement, two-way mixed effects, absolute agreement intraclass correlation coefficients for all Achilles tendon dimensions. A logistic regression analysis was performed to determine how the free Achilles tendon length affected the risk for Achilles tendinopathy while adjusting for age, weight, and height. Finally, the correlation between free Achilles tendon length and thickness was calculated in the combined Achilles tendinopathy cases. Since the sample size is relatively small and some of the data is non-normal, Kendall's Tau was selected as the most appropriate correlation coefficient [47].

## 3. Results

**Figure 3:**
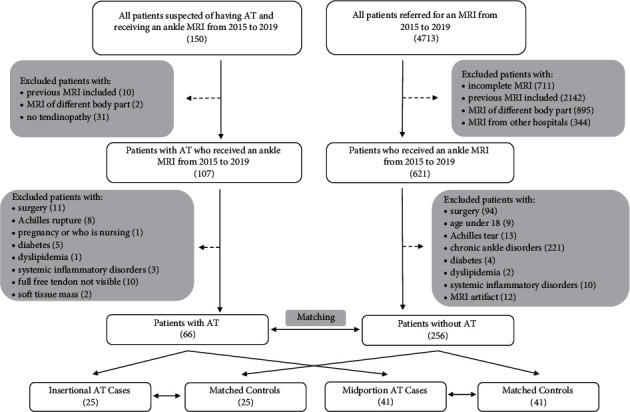
Flow chart of the selection process for Achilles tendinopathy (AT) cases and controls.

**Figure 4:**
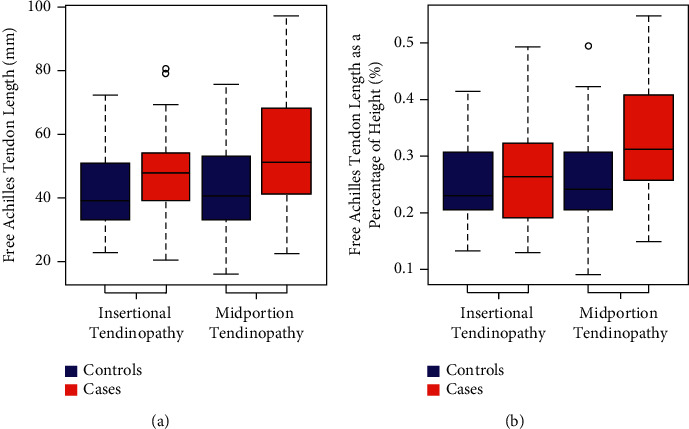
The distribution of (a) free Achilles tendon length and (b) Achilles tendon length as a percent of height between cases and controls. The coloured boxplots represent the interquartile ranges, which contain 50% of individual subjects' lengths. The thick black line represents the median. The whiskers are lines that extend from the box to the highest and the lowest values, except outliers (small circles). A significant difference was found between the midportion cases and controls.

MRI scans of 41 midportion Achilles tendinopathy patients and 25 insertional Achilles tendinopathy patients were selected and matched to control patients scans by sex, age, and weight (Figure 3). There was no significant difference in demographic data between the cases and control groups (Table 1). The thickness and cross-sectional area measurements were significantly larger (*p* < 0.001) in the cases compared to the controls (Table 1). The free Achilles tendon lengths were longer in patients with Achilles tendinopathy compared to controls (Figure 4(a)), and this difference was significant in the midportion Achilles tendinopathy group with median measures of 51.2 (interquartile range 26.9) mm and 40.8 (interquartile range 20.0) mm, respectively (*p*=0.007). In a non-prespecified exploratory analysis, the percent of height was calculated for each free Achilles tendon length value, and these percentages were also found to be significantly higher in the midportion cases compared to controls (Figure 4(b), *p*=0.008). Intra- and inter-rater intraclass correlation coefficients demonstrated excellent reliability (>0.9) regarding all measurements except location of pathology, which demonstrated good inter-rater reliability (0.75–0.9) [48].

### 3.1. Risk of Tendinopathy

Two logistic regressions were performed to investigate the effects of free Achilles tendon length on the likelihood that patients will present with midportion Achilles tendinopathy. Model A adjusted for age and weight. Model B adjusted for age, weight, and height, with fewer participants due to missing data (42 vs. 82). The observations were independent (random pattern on residual vs. order plots). There was a linear relationship between each predictor variable and the logit of the response variable. There were no influential observations (Cook's *D* < 0.5) and no multicollinearity among predictor variables (variance inflation factor <2.5). Non-statistically significant Chi-squared tests indicated both logistic regression models adequately fit the data. The models explained 12.5% and 19.3% (McFadden's *R*^2^) of the variance in Achilles tendinopathy diagnosis, respectively. The results of the logistic regression suggested free Achilles tendon length is a statistically significant predictor of having midportion Achilles tendinopathy. All others held constant, every 1 cm increase in length was associated with 1.53 higher odds of having Achilles tendinopathy using model A (95% confidence interval 1.13 to 2.07, *p*=0.006) and 1.64 higher odds using model B (95% confidence interval 1.13 to 3.15, *p*=0.015). The logistic regression performed on the insertional Achilles tendinopathy data did not reveal significant results (*p*=0.150).

### 3.2. Correlation between Free Achilles Tendon Length and Thickness

Kendall's rank correlation coefficient was computed to assess the relationship between the length of the free tendon and the thickness of the tendon at the thickest section. When analyzing both insertional and midportion Achilles tendinopathy cases combined, there was a moderate positive correlation between the two variables, *r*_*τ*_ = 0.25, *p*=0.003 [49]. Conversely, when looking at the controls, no correlation was found between the two variables, *r*_*τ*_ = −0.01, *p*=0.89.

## 4. Discussion

Increases in the distal free tendon length were associated with higher odds of having midportion Achilles tendinopathy, all others held constant. This suggests that free Achilles tendon length may be a risk factor for Achilles tendinopathy, but a prospective study should be completed in order to make a causal conclusion. This observation has been confirmed by two recent studies. Both found even larger differences between the cases (59.7 mm and 60.0 mm) and controls (38.5 mm and 39.3 mm) [20, 26] However, they did not consider differences in height and weight, so there was the possibility the tendinopathy group was simply taller or heavier. This study also builds on the previous two findings by distinguishing between insertional and midportion Achilles tendinopathy, as risk factors likely differ for these two entities.

The shorter free tendon lengths in this study compared to previous ones may in part be due to the necessary exclusion of 10 cases of Achilles tendinopathy with MRI scans that did not capture the full free tendon. The tendon was long enough that the soleus was not in view and the full free tendon could therefore not be measured. This would result in a potential under-estimation of free Achilles tendon length (i.e., bias) in those with Achilles tendinopathy. This source of bias would be expected to result in a more conservative estimate of the impact of free Achilles tendon length on presence of pathology. Unlike previous study, we adjusted for two potential confounders, height and weight. This removes the risk of longer tendon simply due to taller patient, or higher Achilles tendinopathy risk due to weight. In the Achilles tendinopathy group, there was an association between the length of the free tendon and the thickness of the tendon, a finding not present in the control group. This indicates that longer free Achilles tendons may tend to produce more severe cases of Achilles tendinopathy.

The Achilles tendon is normally posteriorly concave. However, in this study, the free tendon was measured as a straight line, which could have affected the accuracy of the results. Kharazi et al. found that Achilles tendons measured using a straight line underestimated the length by 0.1 to 4.3 mm during the walking gait cycle [50] Since this straight-line technique was used for all patients, both cases and controls, the comparison of the free tendon lengths is justifiable. There was no significant difference in tibiotalar angle between the two groups (Table 1). Furthermore, most of the patients in this study, both Achilles tendinopathy and non-tendinopathy, had their MRI performed with their ankle in a neutral position with an average joint angle of 107°. In this position, the tendon is relatively straight compared to its more curved orientation in plantar flexion [51] The maximum tibiotalar angle was 129°. For all included scans where the tibiotalar angle was above 120° (4 cases, 2 controls), we conducted a non-prespecified exploratory analysis in which the curved-line length of the Achilles was measured and compared to the straight-line length. The maximum difference when comparing the two different length measurement techniques was 0.31 mm, which we consider to have had no or minimal impact on the measurements of free tendon length, which were in the range of 16–98 mm.

This study did not evaluate activity level, which could be a mediating variable. Long free tendons may be more likely to develop Achilles tendinopathy if they are also physically active. Furthermore, longer tendons have been shown to increase running economy due to their ability to store and return a more elastic energy compared to shorter tendons [52–55] Therefore, it is possible that more tendinopathy cases exist with longer tendons simply because these individuals are better runners and therefore run more frequently and intensely. However, these studies measured the full Achilles tendon, not just the free tendon [52–55] When analyzing the sub-tendons individually, Ueno et al. found no correlation between length of the soleus sub-tendon and running economy [53] In fact, elite runners actually have shorter free tendons compared to controls [56] Another study showed that marathon running performance was correlated with soleus volume as well as soleus muscle-to-tendon ratio, indicating that having a lower lying soleus (short free tendon) is actually beneficial to running [57] Furthermore, routine MRI is not able to detect sub-tendons. Future studies should use an MRI protocol using ultrashort TE sequences to be able to visualize collagen fibrillar structure within the tendon. Analyzing the rotation of each sub-tendon could further explain the relationship between tendon length and Achilles tendinopathy.

Knowing that tendon length can influence Achilles tendinopathy risk, it is possible for scientists to make an accurate biomechanical model of Achilles tendinopathy vs. a normal tendon, and better understand the mechanism of injury such as altered energy absorption or stiffness. This information can also help early detection of Achilles tendinopathy. Physicians may be able to palpate, X-ray or ultrasound a patient's ankle to estimate the relative length of the free tendon. This information may make it possible, in future, to direct at-risk individuals towards appropriate preventative strategies methods.

## 5. Conclusion

This study demonstrates that individuals with longer free Achilles tendons have higher odds of developing midportion Achilles tendinopathy, independent of sex, age, weight, and height. [58].

## Figures and Tables

**Table 1:** Descriptive data of case and control groups.

	Insertional Achilles tendinopathy			Midportion Achilles tendinopathy		
	Cases	Controls	*p*-value	Cases	Controls	*p*-value
Sex (*N*)						
Female	17 (68%)	17 (68%)	1^*∗*^	23 (56%)	23 (56%)	1^*∗*^
Male	8 (32%)	8 (32%)		18 (44%)	18 (44%)	
Age (years)	61 (18)	55 (15)	0.323^‡^	55 (17)	52 (12)	0.158^‡^
Height (cm)	163 (11.4)	168 (8.65)	0.579^‡^	170 (23.0)	171 (17.8)	0.795^‡^
Weight (kg)	81.7 (17.2)	78.0 (14.5)	0.303^‡^	83.9 (16.4)	77.1 (15.8)	0.129^†^
Side (N)						
Left	14 (56%)	12 (48%)	0.571^*∗*^	22 (54%)	16 (39%)	0.184^*∗*^
Right	11 (44%)	13 (52%)		19 (46%)	25 (61%)	
Joint angle (°)	110 (9.6)	108 (8.7)	0.529^‡^	106 (7.7)	108 (9.3)	0.193^‡^
Thickness (mm)						
MTJ	7.22 (1.75)	6.44 (1.20)	**0.033 ** ^‡^	7.43 (2.68)	6.11 (1.52)	**<0.001 ** ^†^
TBJ	8.31 (1.93)	5.23 (1.40)	**<0.001 ** ^‡^	5.34 (1.12)	5.06 (1.06)	0.121^†^
Thickest	9.76 (2.61)	6.91 (1.17)	**<0.001 ** ^†^	9.64 (2.87)	6.71 (1.24)	**<0.001 ** ^†^
CSA (mm^2^)						
MTJ	87.5 (19.7)	72.12 (17.9)	**0.003 ** ^†^	99.3 (33.2)	74.7 (21.1)	**<0.001 ** ^†^
TBJ	203 (128)	123 (25.1)	**<0.001 ** ^†^	128 (44.9)	118 (30.6)	0.099^†^
Thickest	191 (110)	86.2 (30.8)	**<0.001 ** ^†^	118 (52.5)	83.5 (27.2)	**<0.001 ** ^†^
LOP (mm)	7.76 (12.7)			35.2 (10.5)		

*Note.* Data were presented as median (interquartile range) or number (percentage). Cases were ankles with Achilles tendinopathy; controls were ankles without Achilles tendinopathy. Statistically significant *p* values were indicated in bold (*p* < 0.05). Height values were missing for 11 pairs in the insertional group and 21 pairs in the midportion group. MTJ values were missing for 4 pairs in the insertional group and 12 pairs in the midportion group. Location of pathology was measured from the calcaneus to the thickest section of the tendon. MTJ = myotendinous junction. TBJ = tendon-bone junction. LOP = location of pathology. CSA = cross-sectional area. ^*∗*^ = data compared using Chi-squared test. ^†^ = data compared using Wilcoxon rank sum test. ^‡^ = data compared using Student's t-test.

## Data Availability

Due to the retrospective nature of the study, we are constrained by our local ethics board and are not able to release individual level data. Data are not available to be shared.
