# Corrigendum to “The Distal Free Achilles Tendon Is Longer in People with Tendinopathy than in Controls: A Retrospective Case-Control Study”

**DOI:** 10.1155/tsm2/9812062

**Published:** 2025-05-28

J. H. Callow, M. Cresswell, F. Damji, J. Seto, A. J. Hodgson, and A. Scott, “The Distal Free Achilles Tendon Is Longer in People with Tendinopathy than in Controls: A Retrospective Case-Control Study,” *Translational Sports Medicine* 2022, no. 1 (2022): 1–9, https://doi.org/10.1155/2022/6585980.

In the article, the authors have identified errors in the abstract and Section 3.1.

In the abstract section:

“MRI-defined free Achilles tendon length is a statistically significant predictor of having midportion Achilles tendinopathy (odds ratio = 0.53, 95% confidence interval 1.13–2.07).”

Should read:

“MRI-defined free Achilles tendon length is a statistically significant predictor of having midportion Achilles tendinopathy (odds ratio = 1.53, 95% confidence interval 1.13–2.07).”

In Section 3.1:

“All others held constant, every 1 cm increase in length was associated with 1.53 higher odds of having Achilles tendinopathy using model A (95% confidence interval 1.13 to 2.07, *p*=0.006) and 1.64 higher odds using model B (95% confidence interval 1.13 to 3.15, *p*=0.015).”

Should read:

“All others held constant, every 1 cm increase in length was associated with 1.53 higher odds of having Achilles tendinopathy using model A (95% confidence interval 1.13 to 2.07, *p*=0.006) and 1.89 higher odds using model B (95% confidence interval 1.13 to 3.15, *p*=0.015).”

We apologize for this error.

